# THE FAST CLINICAL EVOLUTION OF A SPITZ NEVUS: THREE-YEAR FOLLOW-UP OF A CHILD

**DOI:** 10.1590/1984-0462/;2017;35;4;00016

**Published:** 2017-09-21

**Authors:** Maria Cristina Pedrazini, Victor Angelo Martins Montalli, Elemir Macedo de Souza

**Affiliations:** a Faculdade São Leopoldo Mandic, Campinas, SP, Brasil.; b Universidade Estadual de Campinas, Campinas, SP, Brasil.

**Keywords:** Spitz Nevus, Nevus, Nevi and Melanomas, Dermoscopy, Nevo de Spitz, Nevo, Nevos e Melanomas, Dermoscopia

## Abstract

**Objective:**

To report the clinical evolution and handling of a Spitz nevus, from its initial flat feature to becoming an irregular, nodular, reddish lesion.

**Case description:**

Female child, phototype II, with a small congenital nevus on the left lower limb and other sustained small nevi. The patient went through annual clinical and dermoscopic evaluations between the ages of three and seven, period during which the nevi located on the left thigh grew rapidly. The clinical hypothesis was Spitz nevus, with indication of surgical removal with a safety margin and anatomopathological study. Considering patient’s age and clinical/histological aspects, the diagnosis of Spitz nevus was confirmed.

**Comments:**

Initial globular pattern and size under 5 mm upon dermoscopy allowed clinical follow-up. However, onset of hyperchromia and rapid growing of the lesion, along with aesthetic concerns, possibility of trauma in the region, and risk of malignancy at puberty guided the decision of total resection and follow-up for recurrence.

## RESUMO

**Objetivo:**

Descrever a evolução clínica do nevo de Spitz, desde sua característica inicial plana até o aparecimento de uma superfície irregular, nodular e avermelhada e a conduta perante essas alterações.

**Descrição do caso:**

Criança do sexo feminino, fototipo II, com um pequeno nevo congênito na perna esquerda e outros pequenos nevos adquiridos. Paciente passou por avaliações anuais clínicas e dermatoscópicas para controle entre 3 e 7 anos de idade, quando um desses nevos, localizado na coxa esquerda, apresentou crescimento rápido. A hipótese clínica foi nevo de Spitz, com indicação de remoção cirúrgica com margem de segurança e posterior análise anatomopatológica. Considerando a idade da paciente e os aspectos clínicos e histológicos, a lesão foi diagnosticada como nevo de Spitz.

**Comentários:**

Uma lesão de padrão dermatoscópico globular e menor que 5 mm permitia acompanhamento clínico, porém a hipercromia, a estética local, o crescimento rápido, a possibilidade de trauma na região e os riscos de transformação maligna na puberdade nortearam a decisão de remoção total e posterior acompanhamento para monitorar qualquer recidiva.

## INTRODUCTION 

Nevi, also referred to as moles, are either congenital or acquired hamartomas that may present brownish or pinkish pigmentation that blackens by the activity of melanocytic cells, responsible for lesion onset. There being no consensus yet, the hypothesis is that genetic factors and sun radiation can alter the characteristics of such lesions.^1^ Initially small, flat, symmetrical, monochromatic nevi can become dome-shaped, verrucous, irregular, and color-mixed. Nevus lesions must be clinically monitored, and the use of a digital or manual dermoscope increases accuracy for malignancy diagnosis from 46% to the naked eye to 93%. Thus, dermoscopy is the best practice and treatment for more complex lesions (borderline lesions: presenting low malignancy potential, but passive of change if not monitored)^2^ that often require more sophisticated anatomopathological examinations, with use of markers or molecular studies.^3^

Spitz nevus predominates in children and young people. It is named after Sophie Spitz, who first described it in 1948 as juvenile melanoma. When present in adulthood, nevus can be confused with melanoma, so a thorough evaluation by dermoscopy and histological studies are required to rule out the hypothesis of an aggressive lesion.^4^ Spitz nevi are rarely present in the elderly, which suggests that there may be regression with aging.^2^ The strong resemblance to melanoma and the risk of malignant transformation - especially during puberty hormonal explosion in females - make surgical resection and histological examination safer than clinical follow-up alone.^5^

This clinical case aims to demonstrate the clinical evolution of a Spitz nevus in a child.

## CASE REPORT

Female child, phototype II, presenting a small congenital nevus on the left lower limb and other small acquired nevi, underwent annual clinical and dermoscopic examinations between the ages of three and seven for monitoring.

**Figure 1:**
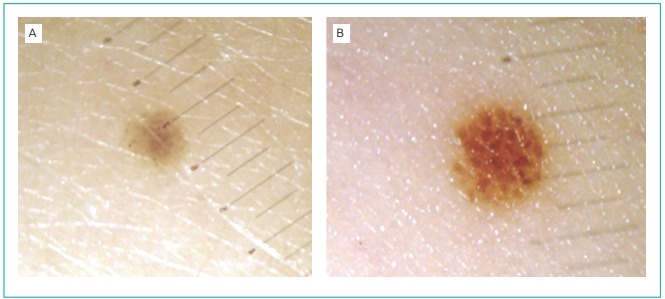
Digital dermoscopy: (A) 1 mm in February 2010; (B) 1.5 mm in March 2011.

**Figure 2:**
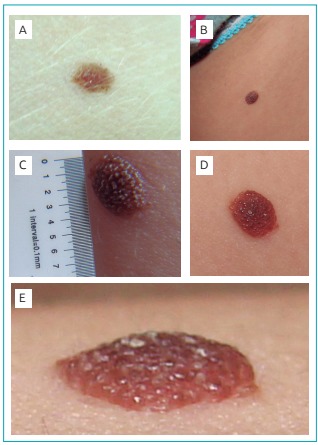
Nevus clinical evolution: (A) 2.0 mm in February 2012; (B-E) 4.5 mm in March 2013.

At three years and seven months of age, during summer, onset of new lesion on the left thigh, close to the groin. Lesion was flat, measuring roughly 1 mm (Figure 1A) and classified as simple acquired nevus. One year later, the lesion was still flat, but showed slight increase in pigmentation. Upon dermoscopy, it was defined as simple nevus with globular pattern measuring 1.5 mm (Figure 1B). At five years and seven months of age, the lesion had increased to 2 mm, became slightly domed and brownish (Figure 2A, and remained as such for approximately nine months. Between six years and five months and six years and eight months of age, after a three-month interval associated with greater exposure to sun during summer, the lesion increased rapidly in extension (from 2 to 4.5 mm) and became more dome-shaped (Figures 2B to 2E). Clinical and dermoscopic progression is shown in Table 1. A new clinical and dermoscopic evaluation was performed and suggested Spitz nevus diagnosis, then surgical removal was indicated.

**Table 1:**
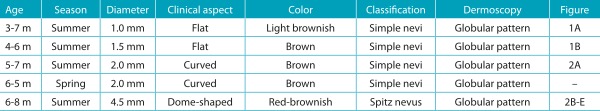
Growth follow-up: left thigh nevus.

**Figure 3:**
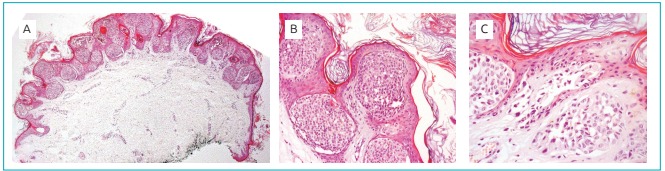
Photomicrograph of Spitz nevus histological sections stained with hematoxylin and eosin (HE) for light optical microscopy: (A) epithelial expansion showing free borders (40x magnification); (B) melanocytic nests (200x magnification); (C) characteristic HE-stained Kamino bodies underlying the epithelium (400x magnification).

Under gas sedation and local infiltration anesthesia, a spindle-shaped incision was made within a 2-mm safety margin, both around the lesion and at depth. The surgery was finished with mononylon suture 7.0. The piece was then fixed in a 10% formalin solution and referred to histological analysis. At macroscopy, the nevus removed from the left thigh measured 0.5 x 0.4 x 0.2 cm and had dome-shaped center, grey-brownish color and slightly bosselated surface, being submitted to histological examination. Upon microscopy, a skin fragment with a slight central bulging was analyzed because of intradermal proliferation. At dermal-epidermal junction, nests of nevus cells with a small amount of melanin pigment in cytoplasm were found. Their nuclei were rounded, with slight volume variation and no atypia, but with some binucleations or multinucleations. Junctional nests of epithelioid pattern were predominant. No fusocellular component were identified, but several Kamino bodies and matured dermal components were present. The overlying epidermis had intense papillomatosis with mild hyperkeratosis, and the surgical margins were free of lesion. There were no signs of malignancy, so the histological findings along with macroscopic study and clinical report suggested Spitz nevus (Figure 3).

## DISCUSSION

Spitz nevus is nowadays treated as a single entity and, given its difficult diagnosis even by histological analysis, it may be classified by its structural characteristics as junctional, intradermal or, more commonly, compound nevus.^6^

Many lesions are indistinguishable from melanoma in adults,^7^ as histological evaluation reveals aggregates of Kamino body, that is, globular structures composed of basement membranes resulting from apoptosis with eosinophil characteristics. Also to be found are epithelioid and/or fusiform cells along with melanocytic nests, often showing atypia with abundant cytoplasm and mitosis, but less than two per mm^2^.^8^ ^,^ ^9^

The most important and peculiar dermoscopic pattern in this lesion is starburst, in 53% of cases, followed by globular (22%) and atypical patterns (25%), the latter being considered a confusing factor for melanoma diagnosis.^2^

Criteria to define the biological behavior of this lesion are still unclear. Some clinical features suggest greater potential for malignancy, such as size larger than 1 cm, tumor extension to subcutaneous tissues, ulcerations, and high mitosis index upon histology.^8^

Spitz nevus occurs more commonly on the face and lower limbs of children and adolescents.^9^ ^,^ ^10^ One of the patterns found upon dermoscopic is globular,^2^ and their growth may be influenced by skin type and sun radiation,^1^ and even by the hormonal action at puberty.^5^ The factors herein described explain the lesion’s behavior in the patient whose case is reported.

Pediatric patients less than 12 years old and presenting with clinical and dermoscopic lesions suggestive of Spitz nevus were followed up in regular consultations for two years, and it showed that some lesions may involute, stop growing or evolve, increasing in size at the same cellular pattern or becoming malignant.^11^

Just like other authors,^12^ ^,^ ^13^ we conclude that typical lesions may be followed up, even though the characteristics found in this case suggest a typical lesion because of its regular borders, size well below 10 mm, non-malignant aspect, and the possibility of regression with aging. The protocol of surgical excision with safety margins, histological analysis and recurrence monitoring was the therapeutic choice to decrease expectations of sudden growth and minimize the risk of trauma in the area, thus improving aesthetics by removing the visible lesion.
